# MANAGEMENT OF A PEDIATRIC LYMPHANGIOMA ON THE BUTTOCKS

**DOI:** 10.51894/001c.122824

**Published:** 2024-08-30

**Authors:** Victoria Qian, Michael Davis, Chaya Pitman-Hunt

**Affiliations:** 1 School of Medicine Wayne State University; 2 Wayne State University School of Medicine, Detroit, MI.

## 13

### BACKGROUND

Lymphatic malformations are congenital non-malignant abnormalities that arise during lymphatic vessel development. They typically present before 2 years of age and are most commonly found in the neck and axillae.

### CASE DESCRIPTION

We report the case of a 14-year-old male who has a 7-year history of a progressively enlarging lymphangioma over his right buttock. The diagnosis was confirmed by initial history and imaging. His presentation is unique in that it did not manifest until he was 7 years old and has since grown to a diameter of 15 cm, covering almost the entirety of his right buttock. This patient was frequently admitted from the emergency department for lengthy hospitalizations due to pain and bacterial infections of the lesion which interfered with educational and personal milestones. Furthermore, the patient expressed that the sensitive position of the lesion negatively impacted his self-esteem and presented a barrier to building intimate relationships. Since presentation, the patient has struggled with adequate pain relief through medical management and sought additional treatment with sclerotherapy, pulsed dye laser therapy, CO2 laser therapy, and shave excisions. He continues to seek optimal management for his condition and is now considering more aggressive options such as surgical and vascular interventions as conservative therapies were unsuccessful.

### DISCUSSION

Although lymphangiomas typically present in the neck and axillae, they can present in other areas of the body, such as the buttock, leading to significant discomfort and decreased quality of life. While many lymphatic malformations remain asymptomatic and only require conservative treatment to resolve cosmetic concerns, caring for patients with symptomatic, complex lymphatic malformations requires a thorough plan involving medical, interventional, and surgical specialist.

